# Development of a self-care scale for women with polycystic ovary syndrome: a methodological approach

**DOI:** 10.3389/fpubh.2025.1656010

**Published:** 2025-10-17

**Authors:** Miok Kim

**Affiliations:** Department of Nursing, College of Nursing, Dankook University, Cheonan, Chungnam, Republic of Korea

**Keywords:** polycystic ovary syndrome, self-care, women’s health, quality of life, factor analysis

## Abstract

**Purpose:**

This study aimed to develop and validate a scale to assess self-care practices in women with polycystic ovary syndrome (PCOS), guided by the middle-range theory of self-care for chronic illness.

**Methods:**

A methodological design was used. Items were generated through literature review and expert input. Content validity was evaluated by experts, and construct validity was tested using exploratory and confirmatory factor analyses (EFA and CFA). Internal consistency was assessed using Cronbach’s alpha. Data were collected from 453 women with PCOS in South Korea.

**Results:**

The final scale consisted of 18 items across five factors, explaining 59.3% of the total variance. Content validity was confirmed, and CFA indicated acceptable model fit. The scale demonstrated high internal consistency (Cronbach’s alpha = 0.90).

**Conclusion:**

This validated scale provides a reliable tool to measure self-care practices in women with PCOS, a common but often overlooked chronic condition. It can be used in clinical and public health settings to promote effective self-management, guide individualized care, and support the development of evidence-based interventions aimed at improving women’s health outcomes.

## 1 Introduction

Polycystic ovary syndrome (PCOS) is a complex and common endocrine disorder that affects up to 13% of women of reproductive age (1). It can occur at any age after puberty, but most women are diagnosed in their 20s or 30s when they visit a healthcare provider due to issues related to pregnancy (2). PCOS is characterized by heterogeneous manifestations, including hyperandrogenism, ovulatory dysfunction, and abnormal ovarian morphology (3). Clinical features such as amenorrhea, oligomenorrhea, hirsutism, obesity, infertility, anovulation, and acne can affect a woman’s identity (4). These symptoms can lead to changes in appearance, potentially causing behavioral disorders and negatively impacting sexual satisfaction, mental health, and overall quality of life (5). As a result, it can negatively affect body image, leading to embarrassment, low self-esteem, and significant psychological distress (6).

In addition, 50–70% of women with PCOS have insulin resistance, which significantly increases the risk of developing other comorbidities such as diabetes, metabolic diseases, hypertension, and dyslipidemia (7). The relationship between insulin resistance, hyperandrogenism, and weight in PCOS is highly complex and has not yet been fully understood (8). Since there is no definitive cure for PCOS, lifestyle modification is recommended as the primary treatment method (9). In particular, the 2018 PCOS Evidence-based Clinical Guidelines emphasize the importance of lifestyle management to improve reproductive, metabolic, and psychological complications (10). Comprehensive management, including diet, physical activity, mood regulation, and treatment adherence, is needed (11). Women with PCOS should establish self-management strategies to prevent disease-related complications, control symptoms, and reduce disease severity, while addressing negative thoughts and seeking support from family and friends (12). Therefore, to effectively promote and accurately measure lifestyle behaviors, it is essential to provide relevant information about PCOS and facilitate its application (13).

Although PCOS is a chronic condition that requires continuous management from adolescence through adulthood, there is a lack of measurement tools that adequately assess self-care practices related to PCOS management and health maintenance. A previous study (14) applied a self-management scale focused primarily on diet and physical activity, but this reflects only a limited aspect of PCOS care. Such tools tend to target general chronic conditions and may overlook PCOS-specific concerns such as menstrual irregularities, hormone-related symptoms, infertility distress, and psychological burdens. Effective PCOS management involves a wide range of strategies, including menstrual cycle regulation, hormone therapy, medication adherence, dietary modification, limiting alcohol and caffeine intake, moderate exercise, metabolic risk management, smoking cessation, stress reduction, psychological support, and the management of infertility and complications (15, 16). Therefore, a more comprehensive and condition-specific tool is needed to accurately assess self-care practices in women with PCOS.

The middle-range theory of self-care of chronic illness (17), derived from Orem’s Theory of Self-Care Deficit, emphasizes the self-care process performed by individuals with chronic illness, rather than interventions from the nursing system. It divides self-care into three components: self-care maintenance, self-care monitoring, and self-care management, which together determine the level of self-care in chronic illness patients. Self-care refers to all activities individuals perform in daily life to maintain and improve their health and well-being. Self-care maintenance involves daily activities to maintain health and prevent deterioration (e.g., balanced diet, regular exercise, proper sleep). Self-care monitoring involves observing and recognizing changes in symptoms (e.g., measuring blood sugar levels, monitoring weight changes). Self-care management includes responding appropriately to changes in symptoms or health status, and consulting healthcare professionals when necessary (e.g., adjusting medication, emergency interventions). Effective self-care plays an essential role in managing disease symptoms and improving quality of life. Therefore, developing tools to assess self-care practices in chronic disease patients is crucial. The middle-range theory of self-care of chronic illness has been verified in studies of Korean adults with hypertension (18) and was the basis for the development of self-care measurement tools in osteoporosis patients (19).

Since PCOS does not have established prevention methods or treatments, it is crucial to follow management guidelines and modify lifestyle to prevent additional complications after diagnosis (20). Therefore, symptom management and health maintenance depend on ongoing self-care by the individual. Self-care is a continuous process of self-directed behavioral change aimed at improving emotional, behavioral, and medical management, with the goal of avoiding disease-related complications, controlling symptoms, and reducing disease severity (21). It includes specific and structured strategies to effectively manage chronic diseases and is a key element in assessing the health management of women with PCOS. In this regard, the middle-range theory proposed by Riegel et al. (17) provides an important theoretical foundation for women with PCOS to develop systematic and continuous self-care strategies for health management. This study aims to develop a measurement tool for assessing daily self-care practices that include all three elements of self-care within the practical context of chronic diseases like PCOS.

## 2 Methods

### 2.1 Research design

This study is a methodological research aimed at developing a self-care scale for women with PCOS and validating its reliability and validity, following the scale development guidelines by DeVellis (22). The process of developing and validating the self-care scale consists of two main stages: the scale development stage and the scale validation stage. Through the scale development process, a preliminary scale was created, and the final scale was confirmed after undergoing validity and reliability testing.

### 2.2 Scale development process

#### 2.2.1 Scale development stage

##### 2.2.1.1 Initial item development

###### 2.2.1.1.1 Literature review

Based on the middle-range theory of self-care in chronic diseases (17), the researcher conducted a literature review to derive a conceptual framework for self-care in women with PCOS. The literature search was performed using domestic and international databases, including PubMed, Cumulated Index to Nursing and Allied Health Literature (CINAHL), Koreanstudies Information Service System (KISS), KoreaMed, Research Information Sharing Service (RISS), and Google Scholar, searching for published academic journals and dissertations. The language was limited to Korean and English. Search terms used in the international databases included “self-care,” “self-management,” and “adherence,” each of which was combined with corresponding MeSH terms using Boolean operators to ensure comprehensive retrieval. After excluding duplicate articles from 210 domestic and 1,521 international papers, the full texts of the articles dealing with the concepts of “self-care, self-management, and adherence in individuals with chronic diseases” and “self-care, self-management, and adherence in women with PCOS” were reviewed.

###### 2.2.1.1.2 Initial item creation

Based on qualitative research on the experiences of women with PCOS and previous studies on self-care in women with PCOS, initial items were developed. These items were reviewed by two experts, and redundant items were deleted. Subsequently, in-depth interviews were conducted with five women diagnosed with PCOS to refine and expand the item pool. A semi-structured interview guide was used to explore their self-care experiences, including daily symptom management, lifestyle practices, emotional coping, and healthcare utilization. Interview data were analyzed using thematic analysis to identify recurring concepts and behaviors. These findings informed the revision and finalization of a total of 57 preliminary items.

##### 2.2.1.2 Response scale and scoring method

To avoid bias toward neutrality during the scale development, a 4-point scale, commonly used in the social sciences, was applied, following Lynn’s (23) method. The response options were: ‘Strongly disagree’ (1 point), ‘Somewhat disagree’ (2 points), ‘Somewhat agree’ (3 points), and ‘Strongly agree’ (4 points). After summing the item scores, a higher total score indicated a higher degree of self-care.

##### 2.2.1.3 Expert content validity testing

In this study, content validity was assessed by calculating the content validity index (CVI, described ahead) based on expert opinions. The preliminary items, developed through a literature review and expert meetings, were reviewed and revised based on in-depth interviews with five women diagnosed with PCOS, and the first round of content validity testing was performed.

###### 2.2.1.3.1 First round of content validity testing

Each item was evaluated for relevance using a Likert scale: ‘Not relevant at all’ (1 point), ‘Somewhat relevant’ (2 points), ‘Quite relevant’ (3 points), and ‘Very relevant’ (4 points). Expert content validity was assessed using the CVI as proposed by Lynn (23), and the item-level CVI (I-CVI) value was considered valid if it was 1.00 for 3–5 experts or 0.78 or higher for 6–10 experts. The first round of content validity testing in this study was conducted with 13 experts. The expert panel consisted of two professors in women’s health nursing, two nursing professors with experience in tool development, one linguistics professor, six clinical nurses and nurse managers caring for women with PCOS, and two obstetricians.

The preliminary items were evaluated based on their relevance to the self-care factors for PCOS patients, as outlined in the middle-range theory of self-care for chronic diseases. Experts assessed whether each item was appropriate, and freely provided suggestions if an item was unclear, irrelevant to the factor, or needed revision. As a result, nine items from a total of 59 were deleted (e.g., “Limit naps to 30 min to avoid affecting nighttime sleep”) due to an I-CVI value of 0.78 or lower. Additionally, some items were revised, such as changing “Drink sufficient water throughout the day” to “Drink an appropriate amount (6–8 cups) of water per day.”

###### 2.2.1.3.2 Second round of content validity testing

Based on the preliminary items refined through the first round of content validity testing, the second round of content validity testing was conducted. The second round involved six experts from the first round: one professor of women’s health nursing, one nursing professor with tool development experience, two nurses caring for women with PCOS, and two obstetricians.

In the second round of content validity testing, the revised preliminary tool from the first round was used, and items with an I-CVI of 0.78 or higher were selected to finalize the preliminary items. As a result, three items were deleted, including “Take prescribed medications for symptoms (e.g., hormonal contraceptives to regulate the menstrual cycle, anti-androgens to reduce excessive hair growth, insulin-sensitizing drugs)” and “Take appropriate medications (e.g., hormone therapy, metabolic improvement agents) at the prescribed times.” Six items were revised, and the final set of 47 items was confirmed.

##### 2.2.1.4 Finalizing the preliminary scale

###### 2.2.1.4.1 Vocabulary revision

A linguistics professor was consulted to ensure that the flow of sentences, the accuracy of the vocabulary, and the clarity of expression were appropriate, and the preliminary tool was refined accordingly.

###### 2.2.1.4.2 Pilot survey

Prior to the main survey, a pilot study was conducted to assess the comprehensibility of the developed scale and the time required to complete the questionnaire. Following the suggestion of Devellis (22), who recommended 20–40 participants for a pilot study, the pilot was conducted with 20 women diagnosed with PCOS. The pilot survey included questions such as “Were there any items that were difficult to understand?”, “Were any items unclear in their expression?,” and “Were there items you felt were not relevant to PCOS self-care?.” Based on the feedback, revisions were made to the items. The pilot study took approximately 10–15 min to complete.

#### 2.2.2 Scale evaluation stage

##### 2.2.2.1 Study participants

A survey was conducted to verify the reliability and validity of the preliminary items. Since PCOS is a common condition in women before menopause, typically under the age of 45 (24), the upper age limit was set at 45 years. On the other hand, in adolescence, anovulation is common, and polycystic ovaries may appear as a normal finding. Additionally, there are limitations in the use of transvaginal ultrasound and unclear androgen normal ranges, making the diagnosis of PCOS difficult in this age group (25). Therefore, the lower age limit for the participants was set at 18 years. Inclusion criteria for the study participants were women aged 18–45 who had been diagnosed with PCOS and were under medical management, understood the purpose of the study, agreed to participate, and were able to respond to the online survey. Exclusion criteria included women who experienced menstrual disorders or hirsutism due to other conditions such as hyperthyroidism or hyperprolactinemia, women with conditions or mental illnesses that could significantly affect quality of life, and women who had difficulty understanding the survey content or self-reporting.

It is recommended that exploratory factor analysis (EFA) and confirmatory factor analysis (CFA) be conducted with different samples (26). The appropriate sample size for EFA is 5–10 participants per item (27), resulting in a range of 235–470 participants. CFA requires at least 200 participants (28). Based on these recommendations, a minimum of 435 participants was required for analysis (235 for EFA and 200 for CFA). To account for a potential 10% dropout rate, the target sample size was calculated as 484 participants (435 ÷ 0.90). A total of 467 participants responded to the survey. After excluding 14 responses that did not meet the inclusion criteria, were incomplete or insincere, or were duplicated based on phone number, 453 responses were included in the final analysis, which met the required minimum. Data analysis was performed using SPSS/WIN 26.0 and AMOS/WIN 26.0 statistical software.

##### 2.2.2.2 Data collection

Data was collected using a self-report questionnaire from one infertility clinic and one online community. The researcher visited the nursing department of the clinic in Gyeonggi Province, South Korea, explained the purpose and methods of the study, and obtained permission for data collection. A recruitment flyer was posted to recruit study participants. Additionally, trained research assistants distributed the study information documents directly to potential participants with the cooperation of the nursing department. The document included the purpose and methods of the study, assurance of voluntary participation and anonymity, the handling of personal information, and how the research data would be processed. Participants who agreed to participate indicated their consent on the online consent form and completed the online survey.

In the PCOS online community, a post explaining the purpose and methods of the study was shared along with a link to the online consent form. Participants read the study information and consent form through the provided link, agreed to the consent form, and then proceeded with the survey. After completing the survey, participants submitted it by clicking the ‘Submit’ button. Participants were informed that they could discontinue their participation at any time, and that any incomplete responses would be discarded.

The online survey was conducted between November 1, 2024, and January 20, 2025, using a structured questionnaire. The survey took approximately 15–20 min to complete.

##### 2.2.2.3 Validity verification

###### 2.2.2.3.1 Construct validity

- Item analysis: The univariate normality of each item, measured on a 1–4 scale, was examined by calculating z-values of skewness and kurtosis (i.e., the statistic divided by its standard error). All z-values fell within ±1.96, indicating that the null hypothesis of non-normality was not rejected at *α* = 0.05, despite the ordinal nature of the items. Raw skewness (< 3) and kurtosis (< 8) statistics also met commonly accepted criteria for approximate normality. Item-total correlations (ITC) of 0.30 or higher were considered acceptable (29). It should be noted that normality is not a requirement for factor analysis; thus, these results do not affect the factor analysis outcomes.
- EFA: To assess the suitability of the data for factor analysis, the Kaiser-Meyer-Olkin (KMO) measure and Bartlett’s test of sphericity were conducted. Based on these results, exploratory factor analysis (EFA) was performed using the principal component method for factor extraction with Varimax rotation, which estimates the factor loadings to identify a parsimonious factor structure capturing the maximum variance in the items. The resulting model was subsequently evaluated through confirmatory factor analysis (CFA).
- CFA: Multivariate normality was assessed using raw skewness (0.52–0.99) and kurtosis (0.02–0.68) values for each item, which met the cut-offs of |skewness| < 3.0 and |kurtosis| < 8.0 (30).

Model fit was evaluated using both absolute and incremental fit indices. In confirmatory factor analysis (CFA), the chi-square test (*χ*^2^) was used to assess the goodness-of-fit of the hypothesized model. The null hypothesis of this test states that the model fits the observed data adequately. A significant chi-square (*p* < 0.05) indicates poor fit, whereas a non-significant result suggests good model fit. Fit indices included CMIN/DF (< 3.00), RMR (≤ 0.05–0.08), RMSEA (≤ 0.05–0.08), CFI (≥ 0.90), TLI (≥ 0.90), and IFI (≥ 0.90) (29, 30, 32).
Items with low squared multiple correlations (SMC) were stepwise removed to improve model fit, and the fit indices were re-evaluated after each modification. The final model was selected based on improved fit and theoretical coherence.

d Convergent and discriminant validity of items: To examine the convergent validity and discriminant validity of the items, standardized factor loadings, Critical Ratios (C. R.), Average Variance Extracted (AVE), Composite Reliability (CR), and the Heterotrait-Monotrait Ratio (HTMT) were assessed. For convergent validity, a standardized factor loading of ≥ 0.50, C. R. ≥ 1.97, AVE ≥ 0.50, and CR ≥ 0.70 were considered acceptable (29), and for discriminant validity, an HTMT value of ≤ 0.85 was applied as the criterion (33).

###### 2.2.2.3.2 Criterion validity

- Concurrent validity: To verify the criterion validity, concurrent validity was assessed by examining the correlation between the newly developed PCOS Self-Care Scale and two established measures: the health-related quality of life (SF-36) and self-efficacy for chronic disease management (SECD-6-K). Pearson’s correlation analysis was used, with correlation coefficients ranging from 0.4 to 0.8 as the expected threshold.

• PCOS is closely related to a decrease in health-related quality of life, not only affecting reproductive function but also overall well-being (34). Self-care promotes self-efficacy as individuals engage in daily health behaviors to improve and maintain their health (35). A high level of self-efficacy is essential for implementing and maintaining lifestyle changes (13). Psychological factors such as low self-esteem and self-doubt can lower self-efficacy, negatively affecting the ability to make lifestyle changes (36). Therefore, this study assessed the concurrent validity of the developed scale based on health-related quality of life and self-efficacy.
• Health-related quality of life was measured using the Medical Outcomes Study Short Form-36 (SF-36) (37). The SF-36 consists of 36 items across 9 domains (physical functioning, social functioning, physical role functioning, emotional role functioning, mental health, vitality, pain, general health, and health change), with scores ranging from 0 to 100. Higher scores indicate better health-related quality of life. The Korean version of SF-36 has demonstrated validity and reliability with a Cronbach’s *α* of 0.93 (38).
• Self-efficacy for chronic disease management was measured using the SECD-6-K, a validated version of the SECD-6 scale (39). Each item was rated on a Likert scale from 1 (“not confident at all”) to 10 (“very confident”), with higher scores indicating higher self-efficacy. The SECD-6 scale had a Cronbach’s *α* of 0.91 in the original development study (39), and a Cronbach’s α of 0.96 in the study by Kim et al. (40).

b Known group validity: Known group validity is assessed when there is an expectation of score differences between groups differentiated by specific characteristics. This method verifies whether the scale can accurately differentiate these groups (41). Given that depression is commonly higher among women with PCOS (42), this study investigated the depression levels in the target population and verified the known group validity of the developed scale. One-way analysis of variance (ANOVA) F tests were conducted to compare mean self-care scores among three depression-risk groups. Significant differences were further examined using Scheffé post-hoc test to determine which groups differed from each other.

• Depression was measured using the Korean version of the Center for Epidemiologic Studies Depression Scale-Revised (K-CESD-R), a revised version of the CESD-R developed by Lee et al. (43). The scale consists of 20 items, with responses ranging from “never” (0 points) to “most of the time” (3 points). The total score is categorized as normal (0–20), at risk for depression (21–40), or high risk for depression (41–60). The reliability of the scale was verified with a Cronbach’s *α* of 0.98 in the original study (43).

##### 2.2.2.4 Reliability verification

Reliability was assessed using Cronbach’s α, the most commonly used method for evaluating internal consistency. A Cronbach’s α of 0.70 or higher was considered acceptable for a new scale (44).

##### 2.2.2.5 Final scale confirmation

Based on the results of the validity and reliability tests, the preliminary scale was finalized as the PCOS Self-Care Scale (PCOS-SC).

### 2.3 Ethical consideration

The study was conducted after obtaining approval from the Dankook University’s Institutional Review Board (DKU 2024-06-007-001). Participants received an explanation of the study and voluntarily signed informed consent before participating. Participants’ anonymity and confidentiality were guaranteed, and the research data was used exclusively for research purposes. Participants were given a small token of appreciation for their involvement.

## 3 Results

### 3.1 General characteristics of the participants

The results of measuring the general characteristics of the study participants, including age, marital status, occupation, religion, education, exercise habits, duration of disease diagnosis, infertility diagnosis, PCOS symptoms, presence of other diseases, smoking and drinking habits, body mass index, and degree of proactivity in disease treatment, are shown in Table 1. Using a random sampling method, data from 254 participants were allocated to the EFA stage, and data from 199 participants were allocated to the CFA stage. A homogeneity test between the two groups showed no statistically significant differences in the general characteristics of the participants between the two groups (Table 1).

**Table 1:** General characteristics of EFA and CFA subjects (*N* = 453).

Characteristics			Total (*n* = 453)	EFA (*n* = 254)	CFA (*n* = 199)	*χ*^2^ (*p*)
			*n* (%)	*n* (%)	*n* (%)	
Age	21 ~ 30		134 (29.6)	68 (26.8)	66 (33.2)	4.747 (0.093)
	31 ~ 40		308 (68.0)	177 (69.7)	131 (42.5)	
	>41		11 (2.4)	9 (3.5)	2 (1.0)	
Marital status	Single		397 (87.6)	225 (88.6)	172 (86.4)	0.476 (0.490)
	Married		56 (12.4)	29 (11.4)	27 (13.6)	
Employment status	Unemployed		61 (13.5)	33 (13.0)	28 (14.1)	0.111 (0.739)
	Employed		392 (86.5)	221 (87.0)	171 (85.9)	
Religion	Non-religious		225 (49.7)	133 (52.4)	92 (46.2)	1.678 (0.195)
	Religious		228 (50.3)	121 (47.6)	107 (53.8)	
Education level	High school or lower		46 (10.2)	29 (11.4)	17 (8.5)	1.011 (0.315)
	College degree or higher		407 (89.8)	225 (88.6)	182 (89.8)	
Smoking status	Non-smoker		383 (84.5)	211 (83.1)	172 (86.4)	1.023 (0.600)
	Former smoker, currently non-smoker		56 (12.4)	34 (13.1)	22 (11.1)	
	Current smoker		14 (3.1)	9 (3.5)	5 (2.5)	
Alcohol consumption	None		256 (56.5)	141 (55.5)	115 (57.8)	1.498 (0.827)
	Less than once a month		66 (14.6)	35 (13.8)	31 (15.6)	
	1–2 times a week		79 (17.4)	49 (19.3)	30 (15.1)	
	1–2 times a week		41 (9.1)	23 (9.1)	18 (9.0)	
Exercise	None		31 (6.8)	18 (7.1)	13 (6.5)	0.054 (0.817)
	Yes		422 (93.2)	236 (92.9)	186 (93.5)	
BMI	≦ 18.5 (Underweight)		78 (17.2)	41 (16.1)	37 (18.6)	2.777 (0.427)
	18.5 ~ 24.9 (Normal weight)		355 (78.4)	199 (78.3)	156 (78.4)	
	25.0 ~ 29.9 (Overweight)		16 (3.5)	12 (4.7)	4 (2.0)	
	≧ 30.0 (Obese)		4 (0.9)	2 (0.8)	2 (1.0)	
Other diseases	None		443 (97.8)	251 (98.8)	192 (96.5)	2.822 (0.093)
	Yes		10 (2.2)	3 (1.2)	7 (3.5)	
Diagnosis period	Within the past year		48 (10.6)	22 (8.7)	26 (13.1)	2.338 (0.505)
	More than 1 year but within 3 years		271 (59.8)	155 (61.0)	116 (58.3)	
	More than 3 years but within 5 years		95 (21.0)	54 (21.3)	41 (20.6)	
	More than 5 years		39 (8.6)	23 (9.1)	16 (8.0)	
PCOS treatment	No treatment		121 (26.7)	71 (28.0)	50 (25.1)	0.456 (0.500)
	Yes, treatment received		332 (73.3)	183 (72.0)	149 (74.9)	
Infertility diagnosis	No		246 (96.9)	190 (96.2)	436 (96.2)	0.582 (0.445)
	Yes		8 (3.1)	9 (4.5)	17 (3.8)	
PCOS symptoms	Emotional changes	No	240 (53.0)	137 (53.9)	103 (51.8)	0.213(0.645)
		Yes	213 (47.0)	117 (46.1)	96 (48.2)	
	Hirsutism	No	287 (62.3)	163 (64.2)	124 (62.3)	0.167(0.683)
		Yes	166 (37.7)	91 (35.8)	75 (37.7)	
	Skin Changes	No	240 (53.0)	127 (50.0)	113 (56.8)	2.061(0.151)
		Yes	213 (47.0)	127 (50.0)	86 (43.2)	
	Weight Gain	No	230 (50.8)	135 (53.1)	95 (47.7)	1.307(0.253)
		Yes	223 (49.2)	119 (46.9)	104 (52.3)	
	Pregnancy-Related Issues	No	355 (78.4)	200 (78.7)	155 (77.9)	0.148(0.827)
		Yes	98 (21.6)	54 (21.3)	44 (22.1)	
	Menstrual Issues	No	198 (43.7)	115 (45.3)	83 (41.7)	0.577(0.447)
		Yes	255 (56.3)	139 (54.7)	116 (58.3)	
Treatment proactivity	Not at all proactive		4 (0.9)	1 (0.4)	3 (1.5)	5.898 (0.207)
	Not very proactive		21 (4.6)	13 (5.1)	8 (4.0)	
	Average		138 (30.5)	84 (33.1)	54 (27.1)	
	Quite proactive		211 (46.6)	108 (42.5)	103 (51.8)	
	Very proactive		79 (17.4)	48 (18.9)	31 (15.6)	

### 3.2 Construct validity verification

#### 3.2.1 Item analysis

The average values for each of the 47 items ranged from 2.85 to 3.24, and the absolute values of skewness and kurtosis for all items were less than 2, confirming that the responses met the normality criteria. The ITC ranged from 0.49 to 0.64, with all values above 0.30, indicating that each item had a significant correlation with the total score and that the scale had consistency and validity.

#### 3.2.2 EFA

EFA was performed on the selected 47 items. The KMO value was 0.91, and the approximate chi-square value for Bartlett’s test of sphericity was 1855.83 (*p* < 0.001), confirming the sample’s suitability for factor analysis. A KMO value greater than 0.6 and a significant Bartlett’s test (*p* < 0.05) indicate that the analysis is appropriate. In the initial analysis, items with communalities below 0.40 were considered for removal. A total of 17 items (items 3, 5, 6, 8, 9, 14, 17, 18, 22, 23, 26, 28, 29, 33, 34, 42, and 43) were excluded based on this criterion. After re-analysis, items 7, 10, 11, 12, 15, 21, 24, and 39, which showed low communalities, were deleted, and after removing these items, items with cross-loadings (items 25, 40, 38) were also removed. Although items 2, 4, 35, 36, and 45 showed moderate cross-loadings (i.e., loading > 0.30 on more than one factor), they were retained due to their theoretical relevance and clinical importance. These items reflected core aspects of self-care in PCOS management that were supported by prior literature and expert review. Removing them would have resulted in the omission of conceptually essential content domains. The final analysis resulted in 20 items that were extracted into five factors, with factor loadings ranging from 0.50 to 0.75 and communalities all above 0.50, explaining 59.3% of the total variance (Table 2). After confirming the factor structure, the factors were named “Routine health management,” “Preventive health management,” “Health problem solving,” “Physical monitoring and regulation,” and “Health information seeking and use” based on the items with high loadings.

**Table 2:** Result of exploratory factor analysis (*N* = 254).

Items	M ± SD	Communality	Factor loading				
			Factor 1	Factor 2	Factor 3	Factor 4	Factor 5
19	3.04 ± 0.85	0.58	0.705	0.220	0.004	0.152	0.088
27	3.03 ± 0.93	0.64	0.656	−0.019	0.213	0.281	0.288
13	3.17 ± 0.92	0.61	0.626	0.346	0.185	0.093	0.224
32	3.12 ± 0.87	0.55	0.590	0.145	0.389	0.118	0.112
02	3.08 ± 0.90	0.50	0.501	0.197	0.408	0.120	0.173
41	3.10 ± 0.84	0.65	0.298	0.715	0.109	0.161	0.123
35	3.11 ± 0.83	0.63	0.095	0.599	0.504	0.049	0.047
30	3.05 ± 0.92	0.59	0.279	0.567	0.069	0.263	0.337
04	2.96 ± 0.89	0.56	0.473	0.511	0.240	0.119	0.061
16	3.15 ± 0.87	0.65	0.013	0.197	0.664	0.365	0.179
37	3.17 ± 0.87	0.57	0.270	0.174	0.660	0.083	0.141
20	3.14 ± 0.88	0.58	0.310	0.015	0.561	0.123	0.395
01	3.16 ± 0.74	0.58	0.128	0.133	0.070	0.729	0.094
31	3.08 ± 0.90	0.62	0.227	−0.060	0.375	0.644	0.104
38	3.10 ± 0.86	0.57	0.052	0.285	0.273	0.618	0.183
36	3.24 ± 0.79	0.55	0.422	0.201	−0.092	0.545	0.159
47	2.85 ± 0.96	0.67	0.124	0.210	0.186	0.091	0.753
46	2.91 ± 0.95	0.65	0.316	−0.049	0.224	0.109	0.695
45	2.90 ± 0.93	0.59	0.010	0.488	0.061	0.212	0.550
44	2.94 ± 0.90	0.55	0.236	0.286	0.113	0.369	0.510
Rotation sums of squared loadings		4.953		4.233	2.325	2.932	4.669
Variance (%)		24.9		21.2	11.6	14.7	23.3
Cumulative variance (%)		24.9		46.1	57.7	72.4	95.7
Kaiser-Meyer-Olkin		0.909					
Barlett’s test of sphericity		*χ*2 = 1855.83, df = 190, *p* < 0.001					

M = mean; SD = standard deviation; EFA = exploratory factor analysis.

Factor extraction method: principal component analysis (PCA).

Rotation method: varimax.

#### 3.2.3 CFA

A model with five factors and a total of 20 items was created and CFA was performed using data from 199 participants, which had not been used in the EFA. The initial model fit indices were *χ*^2^ = 349.81 (*p* < 0.001), CMIN/DF = 2.18, GFI = 0.86, RMR = 0.04, RMSEA = 0.07, IFI = 0.84, TLI = 0.81, and CFI = 0.84. The CMIN/DF (2.18) was considered appropriate (≤ 3.00), GFI (0.80) met the acceptable criterion (≥ 0.80), and RMR (0.07) and RMSEA (0.07) were within acceptable levels (≤ 0.08). However, TLI (0.81) did not meet the acceptable standard (≥ 0.90), indicating a poor fit. To improve the model fit, item 32, which had a low squared multiple correlation (SMC) value of 0.19 (related to symptom management medications such as hormone regulators, metabolic improvement agents, anti-androgens), was deleted. After removing this item, the model fit indices for the remaining 19 items improved to *χ*^2^ = 298.78 (*p* < 0.001), CMIN/DF = 2.10, RMR = 0.04, GFI = 0.87, IFI = 0.86, TLI = 0.83, CFI = 0.86, RMSEA = 0.07. Key indicators such as GFI (0.86) and RMR (0.04) improved. After removing item 1, which also had a low SMC value (0.19) related to weight management and balanced diet planning, the final model with 18 items had fit indices of *χ*^2^ = 258.52 (*p* < 0.001), CMIN/DF = 2.06, RMR = 0.04, GFI = 0.88, IFI = 0.88, TLI = 0.85, CFI = 0.87, and RMSEA = 0.07. Although several fit indices (e.g., GFI = 0.88, CFI = 0.87) did not reach the conventional cutoff of 0.90, the final model was theoretically coherent and demonstrated improved fit after stepwise item deletion. Given the multidimensional and behaviorally grounded nature of self-management—encompassing lifestyle, symptom control, and proactive health behaviors—perfect model fit may be difficult to achieve. Therefore, the revised model with 18 items was considered acceptable for practical use in assessing self-management in women with PCOS. Nonetheless, the less-than-ideal fit indices are acknowledged as a limitation and suggest the need for further refinement in future studies.

#### 3.2.4 Convergent and discriminant validity

Standardized factor loadings were all above 0.50, and the C.R. values were all above 1.97, showing statistical significance. Although the AVE (Average Variance Extracted) values for all paths were below 0.50, the CR (Composite Reliability) values ranged from 0.60 to 0.75, with four of the five factors exceeding the 0.70 criterion. Since the CR value reflects the consistency between items, even when the AVE value is low, a high CR value indicates that the items are reliably functioning. The AVE value is a more conservative criterion, and even when CR ≥ 0.7 and AVE < 0.5, a high CR value supports adequate convergent validity (44). One study (45) suggests that an AVE ≥ 0.4 and CR ≥ 0.6 are acceptable. The third factor did not meet these criteria, but because it measured a core area of self-care, it was retained based on its theoretical importance.

HTMT values are recommended to be below 0.85, and for conceptually close factors, values below 0.90–0.95 are considered acceptable for discriminant validity (33). In this study, most HTMT values were below 0.85, which was within an acceptable range, except for the HTMT value of 0.99 between factors 2 and 3. Although attempts were made to improve the model by removing some items and merging factors, the HTMT indices did not drop below the acceptable threshold across all factors. Additionally, merging the two factors led to a deterioration in the fit indices. When comparing the five-factor model to the four-factor model, the five-factor model showed better fit indices, and although the factors for self-care maintenance, self-care monitoring, and self-care management were conceptually similar, they showed distinct structures. Therefore, the existing five-factor structure was maintained, as it provided better theoretical validity (Table 3).

**Table 3:** Convergent validity of the PCOS self-care scale (*N*=199).

Factors			Standardized factor loadings	Unstandardized factor loadings	SE	C.R.	AVE	CR
1. Routine health management	19. An adequate amount of water (about 6–8 cups) is consumed per day		0.61	1.00	–	–	0.39	0.72
	27. Physical changes such as weight, skin, and hair condition are monitored and managed for health maintenance.		0.58	0.86	0.14	6.10		
	13. A regular lifestyle pattern is maintained to improve sleep quality		0.72	1.21	0.15	7.65		
	02. Meals are eaten at regular times every day		0.58	0.93	0.14	6.35		
2. Preventive health management	41. Regular health check-ups are conducted to prevent the risk of cardiovascular disease and diabetes		0.64	1.00	–	–	0.37	0.70
	35. Regular visits to an obstetrician/gynecologist or endocrinologist are made to assess health status		0.59	0.88	0.12	7.20		
	30. Regular meals and sufficient sleep are maintained to prevent and alleviate fatigue, headaches, or mood changes		0.60	0.87	0.12	6.78		
	04. Regular physical activity is practiced to maintain a healthy weight		0.59	0.87	0.13	6.67		
3. Health problem solving	16. When stressed, emotions are relieved by talking to a trusted person		0.56	1.00	–	–	0.33	0.60
	37. Consultation with medical professionals is sought, and supplements (such as inositol, vitamin D, omega-probiotics, etc.) are taken to improve symptoms		0.57	0.98	0.16	5.84		
	20. Abnormal signs related to the menstrual cycle (e.g., amenorrhea, irregular periods, scanty periods) are monitored, and appropriate action or consultation with a professional is taken when necessary		0.60	0.99	0.16	6.02		
4. Physical monitoring and regulation	31. Lifestyle habits are managed to maintain a regular menstrual cycle, and early visits to a doctor are made when there are changes in the cycle to assess health status		0.65	1.00	–	–	0.43	0.70
	38. Personal health goals (e.g., pregnancy preparation, weight loss, symptom relief) are set, and the execution plan and progress are tracked		0.65	0.99	0.13	7.26		
	36. Hormone treatments prescribed by a doctor are followed to regulate menstrual irregularities, and changes in the menstrual cycle are consistently recorded		0.67	0.87	0.12	7.23		
5. Health Information seeking and utilization	47. Information about changes in symptoms or health status is obtained through support groups, and consultation with professionals is sought when necessary		0.74	1.00	–	–	0.42	0.74
	48. Information on the latest research and treatment options for PCOS is gathered from professionals or media (online or offline resources) and applied to manage symptoms.		0.56	0.74	0.12	6.15		
	45. Programs such as cognitive behavioral therapy are explored to improve negative thought patterns, behaviors, and mental health outcomes		0.69	1.04	0.13	7.86		
	44. When experiencing emotional difficulties such as stress or depression, ways to alleviate these feelings are sought	0.58	0.75	0.11	6.54		

The first item in each factor was set as the reference indicator; therefore, SE and C.R. values are not computed and indicated as “–”.

SE = Standard error; C.R. = Critical ratio; AVE = Average variance extracted; CR = Construct reliability.

### 3.3 Criterion validity verification

#### 3.3.1 Concurrent validity

To evaluate the relationship between the developed tool and external criteria with established validity, concurrent validity was measured using the “Health-Related Quality of Life” and “Chronic Disease Management Self-Efficacy” tools. The correlation coefficient with “Health-Related Quality of Life” was r = 0.36 (*p* < 0.001), and with “Chronic Disease Management Self-Efficacy,” it was *r* = 0.53 (*p* < 0.001), showing moderate correlations, thus confirming the concurrent validity (Table 4).

**Table 4:** Concurrent validity (*N* = 199).

	PCOS-SC (total)	Factor 1	Factor 2	Factor 3	Factor 4	Factor 5
	r (*p*)	r (*p*)	r (*p*)	r (*p*)	r (*p*)	r (*p*)
Health-related QOL	0.36 (<0.001)	0.33 (<0.001)	0.34 (<0.001)	0.31 (<0.001)	0.33 (<0.001)	0.13 (0.069)
Self-efficacy	0.53 (<0.001)	0.44 (<0.001)	0.45 (<0.001)	0.41 (<0.001)	0.33 (<0.001)	0.41 (<0.001)

PCOS-SC = PCOS Self-Care scale.

#### 3.3.2 Group comparison validity

The group comparison validity was tested by comparing self-care levels across three groups based on depression risk. Among the participants, 110 (55.3%) were in the normal group, with a mean self-care score of 3.32 (SD = 0.35). The depression-risk group consisted of 75 participants (37.7%) with a mean self-care score of 2.79 (SD = 0.49), and the high-risk group included 14 participants (7.0%) with a mean self-care score of 2.98 (SD = 0.42). The results showed that the normal group had significantly higher self-care levels compared to the depression-risk and high-risk groups (*F* = 36.213, *p* < 0.001) (Table 5).

**Table 5:** Group comparison validity (*N* = 199).

		*n* (%)	PCOS-SC	F (*p*)
Depression	Normal^a^	110 (55.3)	3.32 ± 0.35	36.213 (<0.001)
	At risk group^b^	75 (37.7)	2.79 ± 0.49	a > b, c
	High risk group^c^	14 (7.0)	2.98 ± 0.42	

PCOS-SC = PCOS Self-Care scale.

### 3.4 Reliability verification

The overall Cronbach’s *α* for the developed scale was 0.90, and the values for the sub-factors were as follows: “Routine health management” 0.76, “Preventive health management” 0.75, “Health problem solving” 0.71, “Physical monitoring and regulation” 0.65, and “Health information seeking and use” 0.74.

### 3.5 Final scale confirmation

The final scale was confirmed and named the PCOS Self-Care Scale (PCOS-SC) for women with PCOS. The PCOS-SC consists of 18 items, with 4 items for “Routine health management,” 4 items for “Preventive health management,” 3 items for “Health problem solving” 3 items for “Physical monitoring and regulation,” and 4 items for “Health information seeking and use.” Each item uses a 4-point Likert scale, ranging from 1 to 4, with a total score range of 18 to 72 points. A higher total score indicates a higher level of self-care practice among women with PCOS.

## 4 Discussion

The EFA identified 20 items across 5 factors explaining 59.3% of the variance. The CFA model was revised to 18 items across 5 factors, with satisfactory fit indices (CMIN/DF = 2.06, RMR = 0.04, GFI = 0.88, IFI = 0.88, TLI = 0.85, CFI = 0.87, RMSEA = 0.07). The 59.3% variance indicates good model fit. Reliability was confirmed with a Cronbach’s *α* of 0.90 for the full scale, and sub-factor reliability ranged from 0.65 to 0.76, with the ‘Physical Examination and Control’ factor showing a lower reliability of 0.65.

To verify criterion validity, the developed self-care scale was correlated with the Health-Related Quality of Life (HRQOL) and Chronic Disease Management Self-Efficacy scales. The self-care scale showed significant positive correlations with both measures, with a moderate correlation (*r* = 0.53) observed with self-efficacy. This suggests that higher levels of self-care are associated with greater self-efficacy, consistent with previous research identifying self-efficacy as a key factor in self-care behaviors (29, 46).

In contrast, the correlation between the self-care scale and HRQOL was relatively weak (*r* = 0.36), warranting further consideration. For women with polycystic ovary syndrome (PCOS), physical functional limitations are generally uncommon, which may reduce the relevance of some items in generic HRQOL measures. However, this finding may also reflect a limitation in the criterion validity of the self-care scale itself. Specifically, the scale may not fully capture psychological or emotional factors that play a central role in determining quality of life for women with PCOS. This limitation suggests that the utility of the scale in predicting or enhancing overall HRQOL may be restricted. Therefore, future studies should consider supplementing the self-care scale with PCOS-specific quality of life measures or revising the scale to better reflect the psychological and social domains that influence well-being in this population.

According to Riegel et al. (17)‘s middle-range theory, self-care consists of self-care maintenance, self-care monitoring, and self-care management. This study reflected this theoretical structure to derive the sub-factors of the tool, resulting in five factors: ‘Routine health management,’ ‘Preventive health management,’ ‘Health problem solving,’ ‘Physical monitoring and regulation,’ and ‘Health information seeking and use.’ These factors align with Riegel et al.’s (17) self-care concept. The naming of the factors was carefully chosen to reflect the concepts they measure. ‘Routine health management’ and ‘Preventive health management’ are similar to the concept of self-care maintenance, while ‘Physical monitoring and regulation’ aligns with self-care monitoring. ‘Health problem solving’ and ‘Health information seeking and use’ are similar to self-care management. However, since these are distinct activities involving symptom management and health information search, they were kept as separate factors to maintain theoretical consistency. While self-care maintenance, self-care monitoring, and self-care management are conceptually distinct, this study decided to maintain the five-factor model to more accurately measure self-care practices. However, it is important to note that there may be potential conceptual overlap between some factors, which should be considered a limitation of the s was identified as a limitation of the study.

Self-care for women with PCOS involves ongoing lifestyle management to maintain both physical and mental health, which can have long-term effects on health outcomes (34). The first factor identified in this study, “Routine health management,” reflects everyday health-maintenance practices such as weight management, monitoring skin and hair changes, and maintaining sleep patterns. This is related to self-care maintenance, which includes actions performed regularly by individuals with chronic diseases to maintain their health (17). PCOS is associated with various physical symptoms such as obesity, metabolic syndrome, skin changes (acne, pigmentation), and hirsutism (10). Managing these symptoms plays a crucial role in preventing disease progression and improving quality of life. Specifically, weight management is emphasized as a key element in managing PCOS (10), and in this study, the item “monitoring and managing weight for health maintenance” was included. Weight gain is related to insulin resistance and can worsen PCOS symptoms, so regular weight monitoring along with dietary and exercise management is necessary (8). Moreover, this factor also includes maintaining a regular lifestyle pattern to improve sleep quality. Research indicates that irregular sleep patterns can lead to hormonal imbalances that regulate appetite, potentially worsening weight gain and insulin resistance, thus negatively affecting PCOS management (47). Therefore, this scale reflects elements that help PCOS women maintain regular lifestyles, which will play an important role in health management and symptom control.

The second factor, “Preventive health management,” encompasses self-care practices aimed at disease prevention and health maintenance, such as “regular visits to gynecologists or endocrinologists for health assessments” and “practicing regular physical activity to maintain a healthy weight.” This aligns with preventive approaches recommended in PCOS management guidelines, such as menstrual cycle regulation and hormone therapy (15). Effective management requires personalized care from a multidisciplinary team comprising endocrinologists, psychologists, nutritionists, and gynecologists (16). However, some studies suggest that focusing too much on weight management may cause women with PCOS to feel neglected or overlook other treatments (48). Nevertheless, weight loss and prevention of weight gain remain essential in PCOS treatment (10), requiring a comprehensive approach that considers both physical and psychological health.

The third factor, “Health problem solving,” captures the specific actions women with PCOS take to actively address health-related challenges. Examples include “talking with a trusted person to relieve stress” and “checking for signs of menstrual irregularities and seeking professional advice when necessary.” This includes proactive actions such as managing physical symptoms or relieving stress, reflecting the importance of individualized plans when symptoms worsen, and utilizing psychological coping strategies to manage stress more effectively (34). Cognitive strategies such as problem-solving and decision-making, along with behavioral strategies like building relationships with health providers and ensuring access to health supplements, can also be incorporated (49). Although this factor showed suboptimal AVE (<0.40) and marginal CR (0.60), it was retained based on its theoretical relevance in PCOS self-care models (17). However, potential construct heterogeneity indicates that the items may not reflect a clearly defined domain, highlighting the need for refinement in future research.

The fourth factor, “Physical monitoring and regulation,” involves self-monitoring of health conditions, tracking changes, setting health goals, and implementing practices. Examples from this study include “following prescribed hormone treatments to manage menstrual irregularities and continuously recording menstrual cycle changes” and “managing lifestyle to maintain a regular menstrual cycle and visiting the hospital for health checks if menstrual cycle changes occur.” Managing lifestyle factors and responding to changes in the menstrual cycle are important criteria for assessing how well women with PCOS recognize and manage their bodies. Effective self-care strategies for PCOS women involve independent monitoring of symptoms and taking personal responsibility for lifestyle choices and medication adherence (34). To promote self-examination and control practices in PCOS women, it is important to go beyond simple health education and emphasize the significance of symptom monitoring. Support from healthcare providers and lifestyle improvement strategies are necessary to help women systematically manage menstrual cycle changes and enable early interventions for long-term health management.

The fifth factor, “Health information seeking and use,” involves collecting information about health conditions or symptom changes and seeking expert help when necessary. This factor includes practices like “obtaining information about symptoms or health conditions from support groups and consulting experts when necessary” and “seeking information on ways to alleviate stress or depression when experiencing emotional difficulties.” This reflects the importance of seeking and utilizing appropriate information to prevent disease-related complications, manage symptoms, and reduce disease severity in PCOS (12). Particularly, strategies that reduce negative emotional states during disease management, as well as emotional support, play a significant role (12), and thus health information search and utilization become an essential part of effective self-care strategies. For women with PCOS, internet use, social media, and online communities are commonly used to search for health information, which can positively influence self-care practices and health behavior changes. However, women with PCOS are at risk of exposure to ineffective or harmful advice by relying on the internet and social media as primary sources of lifestyle information instead of healthcare professionals, which can negatively affect the success of lifestyle changes (50). Therefore, it is important to educate women on how to search for accurate information and provide reliable information through professional intervention.

Although the five-factor model showed superior fit and aligned with the theoretical framework, the high HTMT value between Preventive Health Management and Health Problem Solving suggests a potential empirical overlap. This may indicate that participants do not clearly distinguish between proactive self-care and reactive problem-solving in practice. Accordingly, future research should consider refining item wording or improving conceptual clarity to enhance discriminant validity between these closely related factors. Despite this limitation, the developed scale offers valuable insights into self-care practices for managing PCOS symptoms such as menstrual irregularities, hormonal imbalances, weight gain, and skin changes. It can be used to assess women’s health status and support them in monitoring and managing their symptoms. Moreover, the scale may assist healthcare providers in designing personalized health management strategies. Given that PCOS is a chronic condition requiring lifelong management and is associated with other health risks such as diabetes and pregnancy complications, the significance of this scale lies in its potential to help women better understand and appropriately manage the characteristics and progression of the disease.

## 5 Limitation

This study has limitations, as the sample was limited to women with PCOS receiving care at a single hospital and from a PCOS online community, making it difficult to generalize the results. In particular, recruitment from an infertility clinic and an online community may have attracted participants with higher levels of awareness or motivation for self-care, potentially influencing the study outcomes. Additionally, the study did not consider the broad spectrum of symptoms, treatments, and management approaches for different age groups or stages of the disease, which restricts generalizability. Furthermore, although the structural model showed generally acceptable fit indices (e.g., CMIN/DF = 2.06, RMR = 0.04, RMSEA = 0.07), some indices such as GFI (0.88), IFI (0.88), TLI (0.85), and CFI (0.87) did not reach the conventional threshold of 0.90. While these values may be considered marginally acceptable according to more lenient criteria, they suggest that the model fit is less than ideal. This indicates the need for further model refinement or the exploration of alternative theoretical frameworks in future research. Another limitation is the high HTMT value (0.99) between the two factors—Preventive Health Management and Health Problem Solving—which suggests a potential lack of discriminant validity. Although these constructs are theoretically distinct, corresponding to “self-care maintenance” and “management,” and the five-factor model showed better fit than the four-factor model, the overlap in measurement items indicates the need for future refinement to more clearly distinguish closely related dimensions of self-care. Additionally, AVE values below 0.50 across all factors indicate the need for further examination of measurement error or redundancy.

## 6 Conclusion

This study developed and validated a scale to assess self-care practices in women with PCOS through literature review and in-depth interviews. The scale’s content, construct, and concurrent validity, as well as its reliability, were verified. It evaluates multiple aspects of self-care and can be applied to develop and assess interventions aimed at improving lifestyle and health management in women with PCOS. This study contributes to the theoretical understanding of self-care and offers foundational data to enhance quality of life and disease management. The validated self-care scale for women with PCOS provides nurses with a reliable tool to assess patients’ self-management behaviors. It enables the identification of areas requiring support, allowing for the development of tailored nursing interventions. This scale can contribute to improved patient engagement and long-term health outcomes in PCOS management. However, due to limitations in sample size and geographic scope, further research is needed to confirm its broader applicability and to identify factors that influence self-care behavior and intervention effectiveness.

## Data Availability

The raw data supporting the conclusions of this article will be made available by the authors, without undue reservation.
